# Ankyrin2 is essential for neuronal morphogenesis and long-term courtship memory in *Drosophila*

**DOI:** 10.1186/s13041-023-01026-w

**Published:** 2023-05-16

**Authors:** Silvia Schwartz, Sarah J Wilson, Tracy K Hale, Helen L Fitzsimons

**Affiliations:** 1 School of Natural Sciences, Massey University, Palmerston North, New Zealand; 2 Current Address: Istituto Italiano di Tecnologia, Center for Life NanoScience, Rome, Italy

**Keywords:** Ankyrin repeat, Ankyrin2, ANK3, Histone deacetylase, HDAC4, Memory, Neuron, *Drosophila*, Mushroom body

## Abstract

Dysregulation of *HDAC4* expression and/or nucleocytoplasmic shuttling results in impaired neuronal morphogenesis and long-term memory in *Drosophila melanogaster*. A recent genetic screen for genes that interact in the same molecular pathway as *HDAC4* identified the cytoskeletal adapter *Ankyrin2 (Ank2)*. Here we sought to investigate the role of Ank2 in neuronal morphogenesis, learning and memory. We found that Ank2 is expressed widely throughout the *Drosophila* brain where it localizes predominantly to axon tracts. Pan-neuronal knockdown of Ank2 in the mushroom body, a region critical for memory formation, resulted in defects in axon morphogenesis. Similarly, reduction of Ank2 in lobular plate tangential neurons of the optic lobe disrupted dendritic branching and arborization. Conditional knockdown of Ank2 in the mushroom body of adult *Drosophila* significantly impaired long-term memory (LTM) of courtship suppression, and its expression was essential in the γ neurons of the mushroom body for normal LTM. In summary, we provide the first characterization of the expression pattern of Ank2 in the adult *Drosophila* brain and demonstrate that Ank2 is critical for morphogenesis of the mushroom body and for the molecular processes required in the adult brain for the formation of long-term memories.

**Supplementary Information:**

The online version contains supplementary material available at 10.1186/s13041-023-01026-w.

## Introduction

Histone deacetylase 4 (HDAC4) is a member of the Class IIa family of histone deacetylases, and loss of *HDAC4* is associated with Chromosome 2q37 deletion syndrome [MIM 600,430], the clinical features of which include developmental delay, autistic features and intellectual disability [1–3]. HDAC4 regulates synaptic plasticity and memory formation in several animal models [4–8]; in mice, brain-specific conditional knockout of *HDAC4* results in impaired spatial memory [5] and we previously showed that in *Drosophila*, both RNAi knockdown or increased expression of *HDAC4* in the brain also impairs LTM formation [4].

To gain further knowledge of the molecular pathway through which HDAC4 acts, we previously carried out a genetic enhancer screen in *Drosophila* photoreceptors for genes that interact genetically with *HDAC4* [9] and identified a group of cytoskeletal regulators including *Ank2*. We focused our attention on further investigating *Ank2*, since the altered expression of the human homologue *ANK3* has been associated with a variety of neurodevelopmental disorders including intellectual disability, epilepsy, attention deficit hyperactivity disorder [10], bipolar disorder [11] and autism spectrum disorder [12]. Moreover, *ANK3* single nucleotide polymorphisms have been associated with schizophrenia [13–17] and Alzheimer’s disease [18].

Ankyrins are adapter proteins that link the underlying spectrin-actin cytoskeleton to integral membrane proteins such as ion channels, anion exchangers, signaling proteins and cell adhesion molecules [19–21]. Canonical ankyrins are comprised of an N-terminal membrane binding domain, which contains 24 ANK repeat motifs organized as two anti-parallel α-helices that mediate protein-protein interactions. They also contain a central spectrin-binding domain, a death domain and a less conserved C-terminal regulatory domain [22, 23]. *Drosophila Ank2* displays high similarity to human *ANK3* [10], sharing 57% amino acid identity over the whole protein and 71.2% identity across the ankyrin repeat region. Similarly to *ANK3*, expression of *Ank2* is restricted to neurons and a number of transcript isoforms are also expressed. In the embryonic nervous system, shorter isoforms of Ank2 (Ank2-S) localize to the cell bodies of neurons, whereas medium Ank2-M isoforms (Ank2-M) localize to axons and are essential for viability [24]. A longer Ank2-L isoform containing an additional C-terminal domain localizes to axons and synaptic boutons at the neuromuscular junction. Mutants with disrupted Ank2-L expression display a loss of synapse stability as well as a reduction in the terminal bouton size, disassembly of the presynaptic active zone and retraction of the synaptic microtubule cytoskeleton [25, 26], thus Ank2-L is required for synapse stability and normal morphology at the neuromuscular junction in *Drosophila* larvae.

Despite comprehensive investigation of the roles that Ank2 plays in neuronal development, the role of Ank2 in the adult brain and functional consequences of reduced Ank2 has received little attention. Here we aimed to investigate the role of Ank2 in learning, memory and development of the *Drosophila* brain.

## Methods

### Fly strains

All flies were raised on standard medium on a 12-hour light/dark cycle and maintained at a temperature of 25°C unless otherwise indicated. w[*]; P{w[+ mW.hs] = GawB}OK107 ey[OK107]/In(4)ci[D], ci[D] pan[ciD] sv[spa-pol] (*OK107-GAL4*), w[*];, *P{w[+ mW.hs] = GawB}elav[c155]* (*elav-GAL4*), *P{w + mW.hs = GawB}c739* (*c739-GAL4*), *w*^*1118*^;*P{w + mW.hs = GawB}c305a* (*c305a-GAL4*), *w*^*1118*^;*P{w + mW.hs = GawB}1471* (*1471-GAL4*), *w*^*1118*^; *P{y[+ t7.7] w[+ mC] = GMR16A06-GAL4}attP2* (*R16A06-GAL4*) and *P{w[+ mW.hs] = GawB}3A* (*3A-GAL4*) were obtained from the Bloomington *Drosophila* Stock Center (abbreviated names that are used in the text are shown in parentheses after each genotype). [*]; P{w{+mW.hs] = GawB}NP1131 (*NP1131-GAL4*) was obtained from the Kyoto Stock Center. *P{w + mC = tubP-GAL80ts}10* (tubP-GAL80^ts^), p{MEF2-GAL4.247} (*MB247-GAL4*) and *w(CS10)* strains were kindly provided by R. Davis (The Scripps Research Institute, Jupiter, FL). *P{KK106729}VIE-260B* (*UAS-Ank2* RNAi1, VDRC ID 107369) and *w*^*1118*^;*P{GD12247)v40638* (*UAS-Ank2* RNAi2, VDRC ID 40638) were obtained from the Vienna *Drosophila* Resource Center. *w[1118]; PBac{EGFP-IV}Ank2[KM0104]* (Ank2::GFP, DGRC No. 109758) was obtained from the Kyoto Stock Center. The insertion of GFP was verified using the following primer sets that amplify across the 5’ and 3’ flanking regions of the insertion: Ank2for GGCACAAGCTCCCCGAA and GFPrev ACCACCCCGGTGAACAGCT, product size = 333 bp; GFPfor CGGCATGGACGAGCTGTAC and Ank2Rev CCTAGATGAGCTGATTGCCCAT, product size = 294 bp. *UAS-HDAC4-Myc* has been previously characterised [6]. To generate this construct, *Drosophila HDAC4* was synthesized by Genscript (New Jersey, USA) (nucleotides 461–4216 of NCBI reference sequence NM_132640 with a C-terminal 6x Myc tag) and cloned into NotI and XbaI of pUASTattB. *UAS-Ank2*_*190-946*_*-HA* consists of a 2268 bp N-terminal region of *Ank2* containing the ankyrin repeat region (nucleotides 1123–3393 of *Ank2*, NCBI reference NM_001274607, which corresponds to amino acids 190–946). This construct with a C-terminal 3x HA epitope tag was generated and subcloned into NotI and XbaI of pUASTattB by Genscript. Transgenic flies were generated by GenetiVision (Houston, TX) using the P2 docking site at (3 L)68A4 for *UAS-HDAC4-Myc* and the VK22 docking site at 2R(57F5) for *UAS-Ank2*_*190-946*_*-HA*. All strains were outcrossed for a minimum of five generations to *w(CS10)* flies. A homozygous line harbouring *w(CS10); P{w + mC = tubP-GAL80ts}10* and *P{w + mW.hs = GawB}OK107* (*tubP-GAL80*_*ts*_; *OK107-GAL4*) was generated by standard genetic crosses, as was (*elav-GAL4; tubP-GAL80*_*ts*_), (*c739-GAL4; tubP-GAL80*_*ts*_), (*tubP-GAL80*_*ts*_, *1471-GAL4*), (*tubP-GAL80*_*ts*_; *MB247-GAL4*), (*tubP-GAL80*_*ts*_, *c305a-GAL4*) (*tubP-GAL80*_*ts*_; *R16A06-GAL4*),(*tubP-GAL80*_*ts*_, *NP1131-GAL4*), (*elav-GAL4; Ank2::GFP) and (UAS-Ank2*_*190-946*_*-HA; UAS-HDAC4-Myc).*

### Immunohistochemistry

Whole flies were fixed in PFAT/DMSO (4% paraformaldehyde in 1X PBS + 0.1% Triton X-100 + 5% DMSO) for one hour then brains were microdissected in 1X PBST (1X PBS + 0.5% Triton X-100). Brains were post-fixed in PFAT/DMSO for 20 min and blocked in immunobuffer (5% normal goat serum in 1X PBST) for three hours prior to incubation with rabbit anti-Ank2-L (1:1000, gift from H. Aberle) [25], rabbit anti-GFP (Abcam, ab290, 1:20,000, Antibody Registry Identifier AB_303395), mouse anti-GFP (Sigma Aldrich, 11,814,460,001, 1:200, Antibody Registry Identifier AB_390913), mouse anti-Brp (DSHB, nc82, 1:100, Antibody Registry Identifier AB_2314866), mouse anti-Futsch (DSHB, 22C10,1:20, Antibody Registry Identifier AB_528403), mouse anti-Repo (DSHB, 8D12, 1:20, Antibody Registry Identifier AB_528448) and mouse anti-Fasciclin II (DSHB, Fas 2, 1D4, 1:20, Antibody Registry Identifier AB_528235). Brains were then incubated with secondary antibody (goat anti-mouse Alexa 488 or 555, or goat anti-rabbit Alexa 488, 555, or 647, Sigma Aldrich, 1:500) and mounted with Antifade (2% n-propyl gallate, 90% glycerol in PBS). The monoclonal antibodies anti-Brp (developed by E. Buchner), anti-Futsch (developed by S. Benzer and N. Colley), anti-Repo (developed by C. Goodman), and anti-Fas2 (developed by C. Goodman) were obtained from the Developmental Studies Hybridoma Bank developed under the auspices of the NICHD and maintained by The University of Iowa, Department of Biology, Iowa City, IA. For confocal microscopy, images were captured with a Leica TCS SP5 DM6000B Confocal Microscope and images were processed with Leica Application Suite Advanced Fluorescence (LAS AF) software and Image J (NIH). For quantification of dendrite branch length, the lengths of the primary and secondary branches of all visual system (VS) neurons were manually traced using the SNT program in the ImageJ NeuroAnatomy plugin, which allows branching trace plots to be reproduced from the dendritic arborizations [27]. To ensure all branches were counted including those with fainter GFP signal, the intensity was increased and each branch was followed through the Z stack. After the primary and secondary branch lengths were traced, these measurements were then added together to produce a total sum branch length. Statistical analysis was carried out with the student’s *t*-test with significance set at p < 0.05.

### RT-qPCR

*elav-GAL4* females were crossed to *UAS-Ank2* RNAi1 males to generate progeny in which *Ank2* was knocked down in all neurons; and progeny of *elav-GAL4* crossed to *w(CS10)* served as the control. Total RNA was extracted from *Drosophila* heads from three independent crosses with the RNeasy Mini kit (Qiagen) according to the manufacturer’s instructions. cDNA was synthesized from 1 µg of total RNA with Transcriptor (Roche) as per the manufacturer’s instructions. RT-qPCR was conducted using SsoFast-EvaGreen (BioRad) reaction master on a Lightcycler II 480 instrument (Roche), following manufacturer’s instructions. The following primers were used: *Ank2*for 5’-GGCCGATATGGCACAAAACC-3’, *Ank2*rev 5’TTCTTTCGACGGTGGTACGG-3’, *EF1a48D*for *5*’*-*ACTTTGTTCGAATCCGTCGC-3’, *EF1a48D*rev 5’-TACGCTTGTCGATACCACCG-3’. A 5-fold dilution of cDNA from control flies was used as template to prepare a standard curve to confirm efficiency of the PCR reactions. Relative quantification was conducted using 2^−ΔΔCt^ method, normalizing to the housekeeping gene *Ef1α48D* [28]. *Ank2* expression was reduced to 0.42 ± 0.12 (mean ± standard error) of that of the control, student’s *t*-test t_(12)_ = 4.74, p < 0.001. Only *Ank2* RNAi1 was assessed via RT-qPCR as *elav-GAL4* driven expression of *Ank2* RNAi2 was lethal. *Ank2* RNAi1 and RNAi2 knockdown in the mushroom body with *OK107-GAL4* was also confirmed by immunohistochemistry. Knockdown in the γ lobes of the adult mushroom body with *NP1131-GAL4* and *R16A06-GAL4* was also confirmed following induction of expression via the GAL80ts system by raising the temperature at which flies were housed from 19 °C to 30ºC for 72 h, which were the experimental conditions used in the courtship suppression assay (Fig S1).

### Courtship suppression assay

The repeat training courtship suppression assay was used to assess 24-hour long-term courtship memory. This is an experience-dependent assay in which wild-type male flies that have been previously rejected by a mated female will reduce their courtship behavior towards a new mated female. During mating, the male pheromone cVA is transferred to the female, and the presence of this pheromone on the female causes the male to reduce his courtship towards her. Males that have previously experienced rejection will suppress courtship towards another mated female due to an enhanced response to cVA [29, 30] and this form of courtship memory is termed cVA-retrievable memory [31]. The detailed methodology has been described previously [4, 32, 33]. For training, single virgin males (3–5 days post eclosion) of each genotype (n > 17/genotype) were placed into individual training chambers. A freshly mated wild-type female was placed with each male to be trained, whereas sham control males were housed alone. Over the seven-hour training period, multiple bouts of courtship were observed in the trained group. The female fly was then aspirated from the training chamber and the males were left in their chambers for the 24 h prior to testing. Each trained or sham male fly was then placed into a testing chamber containing a mated wild-type female and was scored for the time spent performing stereotypic courtship behaviors over the ten-minute period. A courtship index (CI) was calculated as the proportion of the ten-minute period spent courting. A mean CI for each group was determined, and from this a memory index (MI) was calculated by the following equation: MI = 1-(CI of each trained fly/mean CI of sham group) (n ≥ 16/group). The MI was measured on a scale of 0 to 1, a score of 0 indicating memory was no different than untrained sham controls. In all experiments, the scorer was blind to the genotype of the flies. For assessment of immediate short-term memory, the training session was reduced to one hour and flies were tested immediately after training. For assessment of learning, the male was placed with a mated female for an hour and the first ten minutes and last ten minutes were scored for courtship behavior. The learning index was calculated as 1-(CI last 10 min/CI first 10 min). For statistical analyses, data were arcsine transformed to approximate a normal distribution and one-way ANOVA with post-hoc Tukey’s HSD test was used to assess significance (p < 0.05).

### Western blotting

Whole cell extracts were prepared from 100 snap-frozen heads by homogenizing in RIPA buffer, followed by centrifugation at 13,000 g for 2 min at 4 °C. Lysates (30 µg) were resolved on 4–20% SDS-PAGE gels (Biorad) and transferred onto nitrocellulose membranes. Membranes were blocked for > 1 h at room temperature in 5% skim milk powder in TBST (50 mM Tris, 150 mM NaCl, 0.05% Tween-20, pH 7.6) then incubated overnight at 4 °C in primary antibody, washed 3 × 5 min in TBST then incubated one hour in secondary anti-mouse, anti-rat or anti-rabbit HRP-conjugated antibodies (GE Life Sciences) as appropriate. Following 3 × 5 min washes in TBST, proteins were detected with Amersham ECL Prime (GE Life Sciences). The following antibodies were used: rabbit anti-GFP (Abcam ab290, 1:10,000); rabbit anti-Myc (Abcam ab9016 1:1,000, Antibody Registry Identifier AB_307014); rat anti-HA (Sigma-Aldrich 11,867,423,001, 1:1,000, Antibody Registry Identifier AB_390918) and mouse anti-α-tubulin (12G10 clone, DSHB, 1:500, Antibody Registry Identifier AB_1157911).

### Co-immunoprecipitation

Whole cell extracts were prepared as per the western blotting method above. Immunoprecipitation (IP) was performed with the Pierce Classic IP Kit (Thermo Scientific) according to the manufacturer’s instructions. Anti-Myc (1 µL, Abcam ab9016) or anti-HA antibody (1 µL, Sigma-Aldrich 11,867,423,001) was incubated overnight with 1 mg of lysate. Following elution in 2x Laemmli buffer, IP samples were processed for SDS-PAGE and western blotting with anti-HA or anti-Myc alongside 30 µg input samples. Anti-α-tubulin (12G10 clone, DSHB, 1:500) was used as a loading control for input samples.

## Results and discussion

### Characterisation of Ank2 expression in the brain

**Fig. 1:**
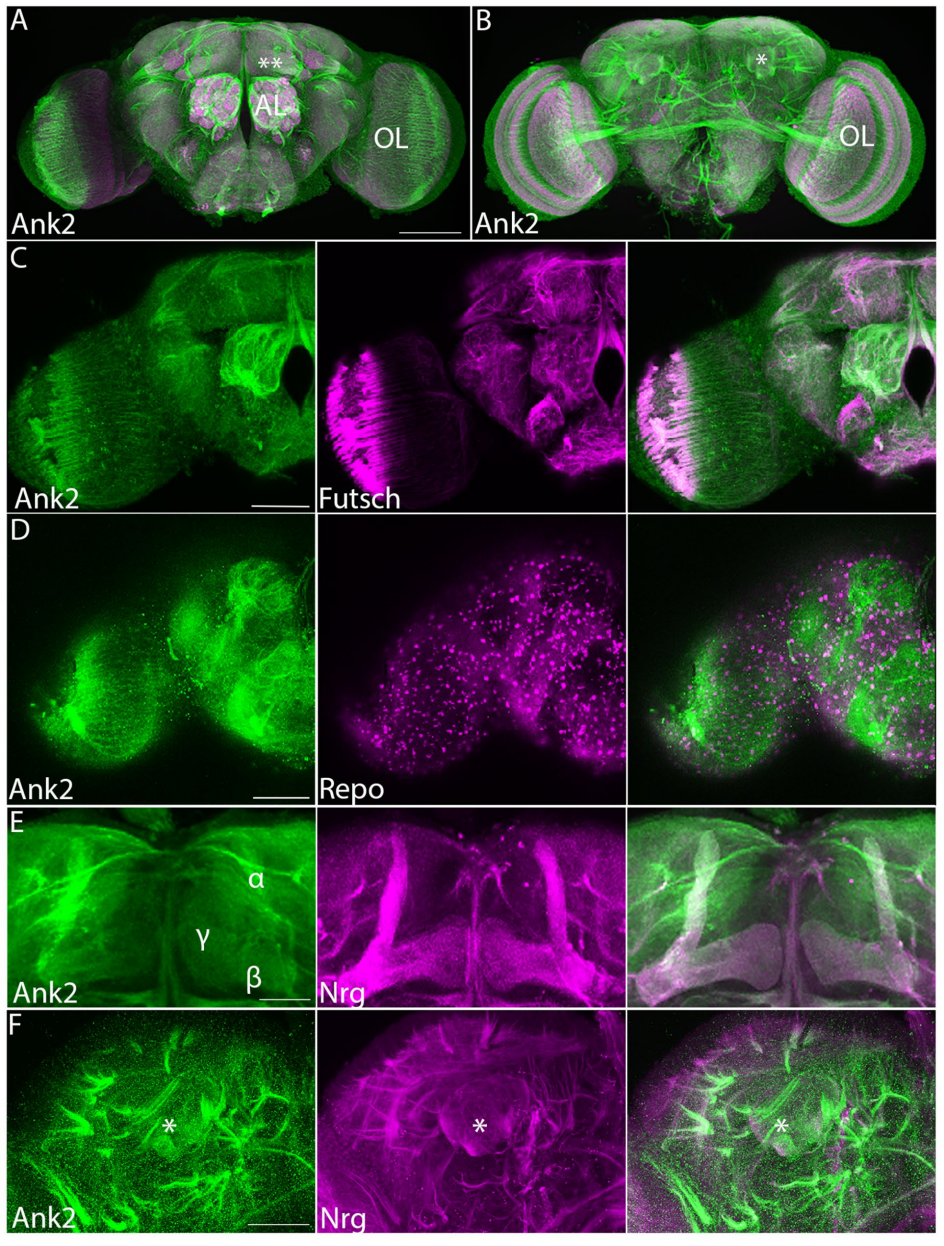
**Expression of Ank2 in the adult brain.** Immunohistochemistry on whole mount wild-type brains indicates widespread distribution of Ank2-L. A-B. Confocal projection of brains labelled with Ank2-L (green) and nc82 (Bruchpilot, magenta) antibodies to highlight the synaptic neuropil. Images are Z-stacks of 1 μm optical sections. (**A**) Anterior confocal projection. AL, antennal lobe, OL optic lobe, **indicates the γ lobe of the mushroom body. (**B**) Posterior confocal projection, *indicates the calyx of the mushroom body. Scale bar = 100 μm (**C-F**). Co-labelled proteins are shown in magenta and labelled in the middle panel. Scale bars = 50 μm. (**C**) Immunohistochemistry with Ank2-L and 22C10 (Futsch) antibodies showing codistribution in neurons, with widespread localization to axon tracts. (**D**) Ank2-L does not codistribute with pan-glial marker Repo. (**E**) Ank2-L colocalizes with Nrg in the mushroom body lobes. (**F**) Ank2-L also codistributes with Nrg in the calyx (asterisk) and surrounding axon tracts.

To date, the expression and localization pattern of Ank2 has been described in the *Drosophila* neuromuscular junction [25, 26] however the expression pattern in the adult brain has not yet been characterized. Immunohistochemistry on whole mount brains with an antibody that detects Ank2-L [25] indicated a broad expression profile with high expression in the optic lobes, antennal lobes, mushroom body and axon tracts throughout the brain (Fig. 1A,B). Colocalization with the axonal marker Futsch [34] confirmed that Ank2-L localizes to axon tracts across the adult brain (Fig. 1C), and a lack of colocalization was observed with the glial marker Reversed Polarity (Repo) [35], confirming its specific neuronal expression pattern (Fig. 1D). Since the mushroom body is a critical structure for memory [36, 37], we examined the expression and subcellular distribution of Ank2 in this brain area in more detail. The intrinsic neurons of the mushroom body are the Kenyon cells, which receive input from the olfactory system [38]. The cell bodies of the approximately 2,500 Kenyon cells are clustered in the posterior dorsal region of the brain and extend their dendrites anteriorly into a globular region known as the calyx. The calyx is organized into an array of microglomeruli, each comprising the large synaptic bouton of a projection neuron from the antennal lobe surrounded by Kenyon cell dendrites [39]. Their axons form a bundled fiber termed the pedunculus and project towards the anterior portion of the brain, forming five distinct lobes; the vertical α and α′ lobes and the medial β, β′ and γ lobes [40, 41] (see Fig. 3F). We examined the colocalization of Ank2-L with the neuronal cell adhesion molecule Neuroglian (Nrg), which has been shown to interact with Ank2 [42, 43]. Nrg is the sole *Drosophila* orthologue of the L1-CAM family of proteins [44], which enables axon guidance through the mushroom body. We confirmed that Ank2-L and Nrg codistribute in multiple axon tracts including the axons of the mushroom body, where both were observed in the α, β and γ lobes (Fig. 1E) and expression in these lobes was further confirmed in Fig S1. Ank2-L was also concentrated in axon tracts surrounding the calyx of the mushroom body (Fig. 1F).

### Ank2 is essential for axon and dendrite morphogenesis

**Fig. 2:**
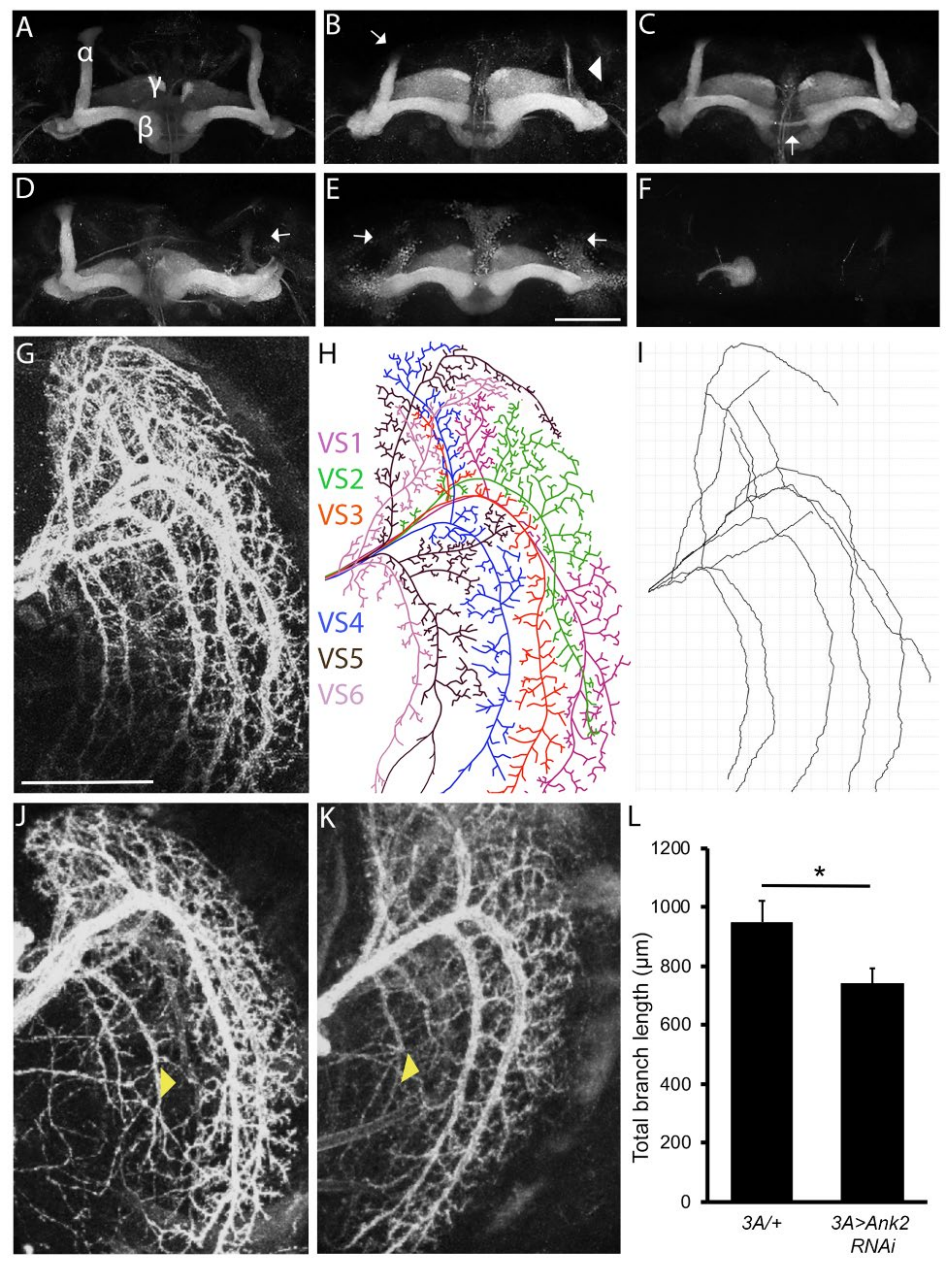
**Reduced Ank2 expression disrupts neuronal development.** (**A-F**) Immunohistochemistry with anti-Fas2 on whole mount brains reveals morphological defects of the mushroom body resulting from knockdown of *Ank2. Ank2* RNAi1 was driven by *elav-GAL4* and *Ank2* RNAi2 was driven by the mushroom body driver *OK107-GAL4*, as *elav-GAL4*-driven expression was lethal. All images are frontal confocal projections through the mushroom body and examples of the range of phenotypes are shown. Scale bar = 50 μm. (**A**) Wild-type mushroom body stained with anti-Fas2 to highlight the α, β and γ lobes. (**B**) Thin α lobe (arrowhead) and a prematurely terminated α lobe (arrow). (**C**) β lobe fusion (arrow). (**D**) Missing α lobe (arrow). (**E**) Both α lobes are missing (arrows). (**F**) None of the lobes have elongated and axon stalling is observed whereby the axons grow in a ball-like structure. (**G-I**) Immunohistochemistry on whole mount brains labelled with GFP to detect Lifeact driven by *3A-GAL4* in LPTCs. All images are confocal projections through the optic lobe of the brain. (**G**) The dendritic arbor of the six neurons comprising the vertical system of LPTCs in a wild-type brain is visualized with anti-GFP. Scale bar = 50 μm. (**H**) Cartoon trace of the confocal micrograph showing the dendritic branching of each of the vertical system neurons. VS1 to VS6 = vertical system neurons type 1 to 6. The branches of each VS neuron type are labelled in a different color, with the main branch, major projecting branches and branchlets shown. (**I**) A manual trace of the main branch and major projecting branches was generated using SNT Tracer (Image J) to calculate the total main and major projecting branch length for each brain (n = 18 to 20 per genotype). (**J,K**) Knockdown of *Ank2* (RNAi1) results in defects in branch extension. Arrowhead in J points to a short VS4 main branch and the arrowhead in K points to a short VS5 projecting branch. (**L**) Total main and major projecting branch length was reduced by knockdown of *Ank2* (student’s *t*-test t_(36)_ = 2.27, p < 0.05)

To investigate whether Ank2 is also required for axon morphogenesis, brains with reduced Ank2 was analyzed via detection of Fasciclin II (Fas2). This cell adhesion molecule is highly expressed in the α, β and γ lobes of the mushroom body [40] and is a commonly used marker to visualize mushroom body lobe architecture (Fig. 2A). Pan-neuronal knockdown of *Ank2* with *Ank2* RNAi1 resulted in a variety of phenotypic defects of the mushroom body, including both thin and missing lobes as well as guidance abnormalities (Fig. 2B-F). GAL4 activity increases at higher temperatures and accordingly we observed more severe defects when the temperature was raised during larval development (Table 1). Knockdown of *Ank2* with a second independent RNAi line also induced a range of mushroom body defects. Efficacy of knockdown was confirmed by immunohistochemistry and RT-qPCR (Fig S1, Methods).

**Table 1:** Frequency of mushroom body phenotypes resulting from knock down of *Ank2*

Genotype	*elav > w(CS10)*	*elav > Ank2 RNAi1*	*elav > Ank2 RNAi1*	*elav > Ank2 RNAi1*	*OK107 > w(CS10)*	*OK107 > Ank2 RNAi2*
Temperature	25 °C	22 °C	25 °C	27 °C	25 °C	25 °C
*n*	40	30	30	16	40	46
Thin α/β or γ lobes	0%	37%	23%	62%	0%	4%
Fused β lobes	0%	13%	10%	0%	0%	0%
Outgrowth/guidance defect	0%	37%	23%	50%	0%	7%
Absent α/β or γ lobes	0%	6%	40%	50%	0%	13%
Total % of brains with defects	0%	73%	73%	100%	0%	35%

As the phenotypes often differ across the two hemispheres of one brain (such as observed in Fig. 2B,D), the percentage of brain hemispheres displaying each phenotype was calculated from the total number of brain hemispheres analyzed for each genotype (n) at 22, 25 and 27 °C. The total percentage of brains displaying defects was also calculated for each genotype. Expression of *Ank2* RNAi1 resulted in a significant number of brains with defects at all temperatures (p < 0.001), and there was an increased number of brains with defects when raised at higher temperatures (27 °C compared to 22 °C, Fisher’s Exact Test p < 0.001). Note that some brain hemispheres displayed thin lobes as well as outgrowth and/or guidance defects therefore the sum of percentage defects may be higher than 100%. Expression of *Ank2* RNAi2 also resulted in significantly more brains with defects than controls at 25 °C (p = 0.0042).

Ank2 has been demonstrated to interact with Nrg through a FIGQY motif within the intracellular domain of Nrg [42]. Nrg is required for normal mushroom body lobe development, whereby the extracellular domain of Nrg mediates cellular adhesion between axons of different mushroom body subtypes for guidance into the pedunculus and lobes. This interaction relies on the presence of Ank2 in either the ingrowing or substrate axon neurons where it is proposed to stabilize the transaxonal Nrg complex [43]. A hypomorphic *Nrg* mutant displayed deficits in axon growth and guidance in the mushroom body [43], which are very similar to those we observed on knockdown of *Ank2*. In addition, Nrg also interacts with a second cytoskeletal adapter protein Moesin (Moe) through a FERM domain in the intracellular domain of L1 CAMs [45], creating a ternary complex between Ank2, Nrg and Moe [43]. Moe is highly expressed in the mushroom body and distributes to the lobes on activation by phosphorylation [33], and likely acts to link the Ank2-Nrg complex to the actin cytoskeleton in mushroom body axons. Interestingly we also previously showed that *HDAC4* interacts genetically with *Moe* [9], and moreover, reduction of *Moe* also shows similar disruption to mushroom body development as with *Ank2* and *Nrg*, with defects in axon elongation and guidance [33, 43]. Together these data support the evidence for a functional relationship between Ank2, Nrg and Moe in mushroom body development.

During *Drosophila* embryonic and larval stages, *Ank2* mutants exhibit reduced dendritic branching, and in *Drosophila* dopaminergic neurons, knockdown of *Ank2* results in decreased dendritic branching points, leading to a reduced total branch length and a lack of branching complexity [27]. To that end, we investigated whether Ank2 is required for dendrite morphogenesis in the adult *Drosophila* brain. As branching and elongation of Kenyon cell dendrites are difficult to visualize, we instead examined lobular plate tangential cells (LPTCs) of the visual system. The LPTCs are a group of six visual system (VS) interneurons in the optic lobe that provide an ideal model system for investigating dendrite growth and branching as they display stereotypical dendritic arborization [39]. Each VS neuron projects a main primary branch with one or more major branches that project anterior or dorsally from the main branch [46]. Individual dendrites are easily visualised via expression of Lifeact, a GFP-fused F-actin binding peptide [47] with the *3 A-GAL4* driver [46] (Fig. 2G), and branch length can be traced and quantified (Fig. 2H, I). The characteristic dendritic branching of the six neurons was disrupted by expression of *Ank2* RNAi1 with severely reduced main and major projecting branch lengths of individual VS neurons (Fig. 2J, K) and a significant decrease in the total branch length for VS1-6 neurons (Fig. 2L). These data suggest that wild-type levels of Ank2 are required for both axon branching, guidance and elongation as well as extension of main and major projecting dendritic branches.

### LTM requires Ank2 expression in the γ lobe of the mushroom body

We next assessed whether Ank2 was required for learning and/or memory with the repeat training courtship suppression assay. This test evaluates the memory of a male following exposure to an unreceptive mated female. Following this failure of mating, a male suppresses his courtship activity towards mated females to which he is subsequently presented. After seven hours of training, males form a stable LTM that lasts for at least 24 h [4, 30, 32]. After this time, each male is placed with a new freshly mated (unreceptive) female and a courtship index is calculated by dividing the amount of time each male spends courting by the total duration of the observation period. A memory index is calculated by comparing the time a trained male spends courting to that of a sham male. A score of zero indicates that memory is impaired and no different from untrained sham controls, whereas a higher memory index indicates intact memory. This form of courtship memory has been recently described as cVA-retrievable memory to differentiate from the associative memory formed when virgin females are used for testing, which uses different circuitry for memory retrieval [31].

**Fig. 3:**
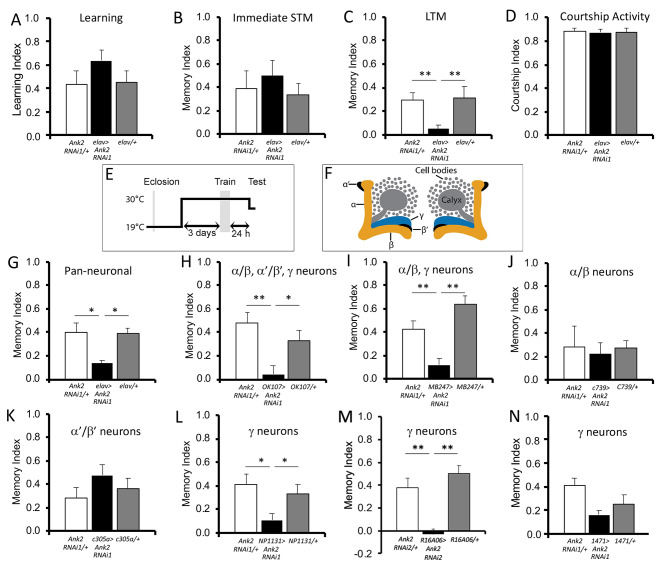
**Ank2 is required for long-term memory.** Learning and memory were assessed with the courtship suppression assay. The controls included in each assay are the GAL4 driver (plus *tubP-GAL80ts* where indicated) crossed to *CS*, and UAS-*Ank2* RNAi1 crossed to *CS*, such that the progeny are heterozygous for either the driver or the RNAi. (**A-D**) *elav-GAL4* and UAS-*Ank2* RNAi1 flies were crossed to achieve pan-neuronal knockdown of *Ank2* in progeny. (**A**) Learning was unaffected by knockdown of *Ank2* (ANOVA, F_(2,47)_ = 0.002, p = 0.252). (**B**) Immediate memory was also unaffected (ANOVA, F_(2,45)_ = 0.044, p = 0.819). (**C**) *Ank2* knockdown impaired long-term memory (ANOVA, F_(2,60)_ = 7.31, p < 0.001; *post-hoc* Tukey’s HSD, **p < 0.01). (**D**) Courtship activity was not impaired by pan-neuronal knockdown of *Ank2* (ANOVA, F_(2,51)_ = 0.14, p = 0.870). (**E-N**) Knockdown of *Ank2* in the adult mushroom body impairs LTM. *Ank2* was knocked down in specific regions of the brain by crossing *Ank2* RNAi to the indicated driver line and *tubP-Gal80ts*. (**E**) Schematic diagram depicting the induction of *Ank2* RNAi expression in the adult mushroom body. Expression was restricted to the adult brain by raising flies at raised at 19°C, at which temperature GAL80 represses GAL4. After eclosion, when flies were 3–5 days old, the temperature was raised to 30°C for 72 hours, after which training commenced. At this temperature GAL80 is inactivated, allowing GAL4 to induce transgene expression. Twenty-four hours after training, the flies were equilibrated to 25°C for one hour prior to testing. (**F**) Schematic diagram labelling the lobes of the mushroom body in which *Ank2* was knocked down. (**G**) Pan-neuronal knockdown of *Ank2* in the adult brain impaired long term memory (ANOVA, F_(2,54)_ = 0.317, p < 0.01; *post-hoc* Tukey’s HSD, *p < 0.05). (**H**) Similarly, memory was also impaired when knockdown of *Ank2* was restricted to the mushroom body (ANOVA, F_(2,52)_ = 0.922, p < 0.001; *post-hoc* Tukey’s HSD, **p < 0.01, *p < 0.05). (**I**) When knockdown was restricted to the α/β and γ neurons of the mushroom body, long-term memory was still disrupted (ANOVA, F_(2,51)_ = 0.923, p < 0.0001; *post-hoc* Tukey’s HSD, **p < 0.01). (**J**) Reduction of *Ank2* in just the α/β neurons had no significant effect on long-term memory (ANOVA, F_(2,41)_ = 0.025, p = 0.819). (**K**) There was also no impairment when *Ank2* was reduced in the α’/β’ neurons (ANOVA, F_(2,51)_ = 0.122 p = 0.372). (**L**) *Ank2* is required in the γ lobes as knockdown with *NP1131-GAL4* impaired LTM (ANOVA, F_(2,46)_ = 0.312, p < 0.01; *post-hoc* Tukey’s HSD, *p < 0.05). (**M**) A second γ lobe driver *R16A06-GAL4* crossed to *Ank2* RNAi2 also impaired LTM significantly (ANOVA, F_(2,33)_ = 18.57, p < 0.0001, post-hoc Tukey’s HSD, **p < 0.01). (**N**) A weaker γ lobe driver *1471-GAL4* also reduced LTM, however this was not quite significant (ANOVA, F_(2,59)_ = 0.210, p = 0.056)

Learning and immediate short-term memory (STM) were unaffected by pan-neuronal knockdown of *Ank2* with *elav-GAL4* (RNAi1, Fig. 3A,B). Pan-neuronal knockdown of *Ank2* during development resulted in a significant and severe loss of LTM formation compared to control genotypes (Fig. 3C). This was not due to an effect on courtship behavior as sham males of each genotype all spent approximately the same percentage of time courting (87 to 89%, Fig. 3D).

Long-term courtship memory is dependent on an intact mushroom body, therefore the memory impairment could be a result of developmental deficits resulting from decreased *Ank2* expression. To allow assessment of the role of Ank2 specifically in adult memory processes, knockdown of *Ank2* was restricted to the mature brain with GAL80ts, a temperature sensitive inhibitor of GAL4 activity [48] (Fig. 3E). Flies were raised at the permissive temperature of 19 °C at which GAL80ts is active. Seventy-two hours after eclosion, male flies from the F1 progeny were transferred to 30 °C to inactivate GAL80ts and thus induce RNAi expression. After three days, males were tested in the courtship suppression assay. Adult-specific knockdown of *Ank2* in all neurons with an inverted repeat hairpin that targets all long isoforms of *Ank2* mRNA (RNAi1) resulted in impairment of LTM formation (Fig. 3G) and when knockdown of *Ank2* was restricted to the adult mushroom body (Fig. 3F) with *OK107-GAL4*, this impairment remained (Fig. 3H). Ank2 was also identified in an RNAi-based screen for genes required for three-hour aversive olfactory memory via pan-neuronal knockdown, [49], suggesting that it is required not just for courtship memory but may be fundamental to different memory pathways.

The three Kenyon cell subtypes are structurally distinct with individually identifiable transcriptomes [50] and have distinct roles in learning and memory [30, 51], thus we next investigated whether there was a differential requirement for Ank2 in specific mushroom body subtypes by restricting expression of *Ank2* RNAi to each subtype individually. Expression with the α/β and γ neuron driver *MB247-GAL4* abolished LTM formation (Fig. 3I). Knockdown in the α/β neurons or α’/β’ neurons did not significantly alter LTM (Fig. 3J,K), whereas knockdown in γ neurons with *NP1131-GAL4* prevented LTM formation (Fig. 3L). An additional γ lobe driver *R16A06-GAL4* also demonstrated reduced LTM upon expression of a second independent RNAi line that also targets all long forms of Ank2 (Fig. 3M). We also tested a third γ lobe driver *1471-GAL4*, which did not quite impair memory to significant levels (p = 0.056, Fig. 3N), likely because this is a much weaker driver than *NP1131-GAL4* [52] and *R16A06-GAL4* [53]. Efficient knockdown following 72 h of RNAi induction in the adult γ lobe with both *NP1131-GAL4* and *R16A06-GAL4* was confirmed by immunohistochemistry (Fig S1). Taken together these data show that in the adult brain, wild-type levels of Ank2-L are required in the γ lobes for normal LTM formation.

Output from γ neurons is required for short-term courtship memory [54] and knockdown of *Ank2* in γ neurons during mushroom body development was recently found to result in a shortened axon initial segment in third instar larval brains [55]. In light of the severe mushroom body defects we observed on knockdown of *Ank2*, and that Ank2 has been implicated in synapse stability [24, 25], it is unsurprising that memory would be impaired. However a specific deficit in 24-hour LTM was still observed when knockdown of *Ank2* was restricted to mature neurons of the mushroom body, and subsequent testing of GAL4 drivers that restrict expression to specific mushroom body subtypes revealed that knockdown in just the γ neurons was sufficient to impair memory. This is consistent with current models of the circuitry that facilitates cVA-retrievable memory [31, 54], which involves activation of aSP13 dopaminergic neurons that innervate the γ5 compartment at the tip of the γ lobe. This results in increased synaptic transmission from γ neurons to glutamatergic M6 output neurons, which themselves feedback to innervate aSP13 neurons to form a recurrent activation loop [54, 56]. LTM requires a later reactivation of aSP13 neurons, which is dependent on sleep [57]. Expression of epigenetic regulators required for cVA-retrievable memory, including Rpd3 [32], HDAC4 [4] and G9a [58] has also been pinpointed to a requirement in only γ neurons. Similarly, the cytoplasmic polyadenylation element-binding protein Orb2 is required for translation of synaptic mRNA during LTM consolidation in the γ lobe [59]. While it is clear that Ank2 is required in the γ lobe, it should be noted that the lack of phenotype resulting from knockdown in the α or β lobes does not preclude the possibility that Ank2 may also play a role in these neurons during LTM formation if the degree of knockdown was not sufficient to deplete Ank2 below a level required for normal function.

We previously showed that overexpression of *HDAC4* in γ neurons also impaired the formation of long-term courtship memory [9]. A primary reason to focus on Ank2 was the fact that HDAC4 contains a conserved PSLPNI ankyrin repeat motif which human HDAC4 has been shown to physically bind to the ankyrin repeat region of both regulatory factor X associated ankyrin containing protein (RFXANK) and ankyrin repeat family A member 2 (ANKRA2) [60, 61].We investigated whether *Drosophila* HDAC4 and Ank2 also interact through this motif, however we did not detect a physical interaction via co-immunoprecipitation. The impact of *HDAC4* overexpression on *Ank2* expression was also examined via an Ank2::GFP protein trap in which *GFP* is inserted into first intron of the endogenous *Ank2* gene. A ~ 75 kDa band detected via western blotting likely corresponds to a very small isoform of Ank2, and was used as a read out for transcriptional regulation of *Ank2* by HDAC4. No changes in protein levels were observed (Fig S2). Since there are multiple isoforms of *Ank2* mRNA and some have a transcription start site downstream of the 1st intron, we cannot rule out alterations to expression of larger isoforms. We examined a genetic interaction in long-term memory by coexpressing *Drosophila HDAC4* and *Ank2* RNAi along with GAL80ts in the adult mushroom body. Expression was partially induced in mature males by raising the temperature to 24 °C, which we have previously determined to drive approximately 50% of maximum expression [9]. At this temperature, there was insufficient expression of *Ank2* RNAi to impair memory, but when combined with *HDAC4*, memory was abolished (Fig S2). This is suggestive of a genetic interaction, however *HDAC4* expression alone was still sufficient to impair memory, although not completely abolish it. Taken together, these data combined with the similarities in phenotypes with regard to the roles in the γ lobe of the mushroom body and axon morphogenesis, it is possible they may act in the same molecular pathways that regulate these processes, however the nature of this potential interaction is yet to be elucidated.

## Electronic supplementary material

Below is the link to the electronic supplementary material.

Fig S1. Characterisation of *Ank2* knockdown in the adult mushroom body. Inverted repeat hairpins for RNAi knockdown of *Ank2* were expressed in in the mushroom body with *OK107-GAL4*. A. Confocal projection of brains labelled with Ank2-L (green) are shown, which are co-labelled with Fas2 (magenta), to highlight the mushroom body. Since *Ank2* knockdown results in defects in lobe morphogenesis in some cases (Table 1), brains were selected in which the α and β lobes were intact in order to verify knockdown. Images in the three left columns are Z-stacks of 1 μm optical sections through the mushroom body. *Ank2* is detected in the α, β and γ lobes of the mushroom body of control (*OK107*/+) brains. Short arrows point to the tips of the α lobe, the longer thinner arrow points to the γ lobe, and the arrowhead points to the β lobe. Expression in these lobes is absent in *OK107>Ank2* RNAi1 and *OK107>Ank2* RNAi2 mushroom bodies. Scale bar = 50 μm. As the expression in the mushroom body is somewhat obscured by other axon tracts, the three right columns show single 1 μm sections through the α lobe (outlined by a dashed line), which confirm knockdown with both *Ank2* RNAi1 and RNAi2. Scale bar = 25 μm. B. The expression patterns of the γ lobe drivers *R16A06-GAL4 *and *NP1131-GAL4* driving CD8::GFP, a plasma membrane-targeted GFP which allows for visualization of GFP in neuronal processes, confirm co-distribution with Ank2 in the γ lobe. The larger images are maximum projections through the mushroom body that show the expression patterns of each driver, with robust expression in the γ but not the α and β lobes (scale bar = 50 μm). Single 1 μm optical sections through the γ lobe labelled with Ank2 (green) and GFP (magenta) show expression of Ank2 in the γ lobes (scale bar = 25 μm). C. Knockdown with *R16A06-GAL4* and *NP1131-GAL4* reduces expression of Ank2 in the γ lobe. Ank2 staining appears overexposed in the Z-projections as it was optimized for detection of the γ lobe in the controls and the settings maintained for all brains. The GAL80ts system was used to knock down *Ank2* in the adult γ lobe by raising flies at 19°C and then increasing the temperature to 30°C in three day old adults for 72 hours, which are the conditions used in the memory assay. Expression is clearly detected in the tips of the α lobes (arrow) as seen in the Z-stack projections (top row, scale bar = 50 μm) but is reduced in the γ lobe, which is outlined by a dashed line in the 1 μm sections (bottom row, scale bar = 25 μm). Two representative images are shown for each RNAi.

Fig S2. Ank2 does not bind HDAC4 nor is its expression regulated by HDAC4. A. Domain structure of HDAC4 showing binding sites conserved between *Drosophila* and human HDAC4. The amino acid sequence of the region containing the PSLPNI ankyrin repeat motif that binds RFXANK and ANKRA2 in human HDAC4 is shown, with the corresponding amino acid sequence in *Drosophila* HDAC4. NLS, nuclear localisation sequence, NES, nuclear export sequence. Ps circled in black are serine residues that when phosphorylated provide binding sites for 14-3-3 mediated nuclear export. B,C. Co-immunoprecipitation of Ank2_190-946_-HA and HDAC4-Myc from whole cell lysates of fly heads expressing *elav-GAL4* driven *UAS-HDAC4-Myc* and/or *UAS-Ank2*_190-946_-HA, as indicated with either anti-Myc or anti-HA. The estimated size of HDAC4-Myc is 144 kDa and Ank2_190-946_-HA is 95 kDa. Both blots were probed with anti-tubulin as a loading control. Input samples = 30 μg. B. Following immunoprecipitation with anti-HA, HDAC4-Myc was not detected upon probing with anti-Myc. C. In the reciprocal experiment, flies expressing Ank2_190-946_-HA; HDAC4-Myc were subjected to IP with anti-Myc, however Ank2_190-946_-HA was not detected upon probing with anti-HA. D. The effect of HDAC4 overexpression on the expression of Ank2 was examined. Ank2::GFP is a protein trap in which an exogenous GFP exon is inserted in frame into intron 1 of the endogenous Ank2 gene [62], resulting in GFP-tagging of a short isoform of Ank2 of approximately 75 kDa. *elav-GAL4; Ank2::GFP* flies were crossed to *w(CS10)* and *UAS-HDAC4-Myc* flies and whole head lysates of progeny were generated for western blotting. Samples were processed from four independent crosses. Blots were probed with anti-Myc to verify expression of HDAC4-Myc, and anti-GFP to determine whether the amount of Ank2::GFP normalized to tubulin is altered in the presence of HDAC4. E. There was no significant change in the level of Ank2::GFP on expression of HDAC4-Myc. F. *MB247-GAL4;tubPGAL80ts* was used to drive expression of *HDAC4* and *Ank2* RNAi1 individually as well as together in the adult mushroom body. Flies were raised at 19°C until after eclosion and expression was induced to approximately half maximal expression by housing at 24°C for 72 hours. At this temperature, partial knockdown of *Ank2* did not impair 24 hour courtship memory, however in combination with half maximal expression of *HDAC4*, memory was reduced to zero (ANOVA, F(_4,66_)=0.0212, p<0.01; *post-hoc* Tukey’s HSD, *p<0.05).

## Data Availability

The datasets supporting the conclusions of this article are included within the article.

## List of abbreviations

HDAC4: Histone Deacetylase 4
MI: Memory Index
CI: Courtship Index
Ank2: Ankyrin2
Nrg: Neuroglian
LTM: Long Term Memory
STM: Short Term Memory
LPTCs: Lobular Plate Tangential Cells
Moe: Moesin
VS: Visual System
