# Supplementary figures and images for: Ankyrin2 is essential for neuronal morphogenesis and long-term courtship memory in Drosophila
Source: Mol Brain. 2023 May 16;16:42. doi: 10.1186/s13041-023-01026-w (PMC10186683; doi:10.1186/s13041-023-01026-w)

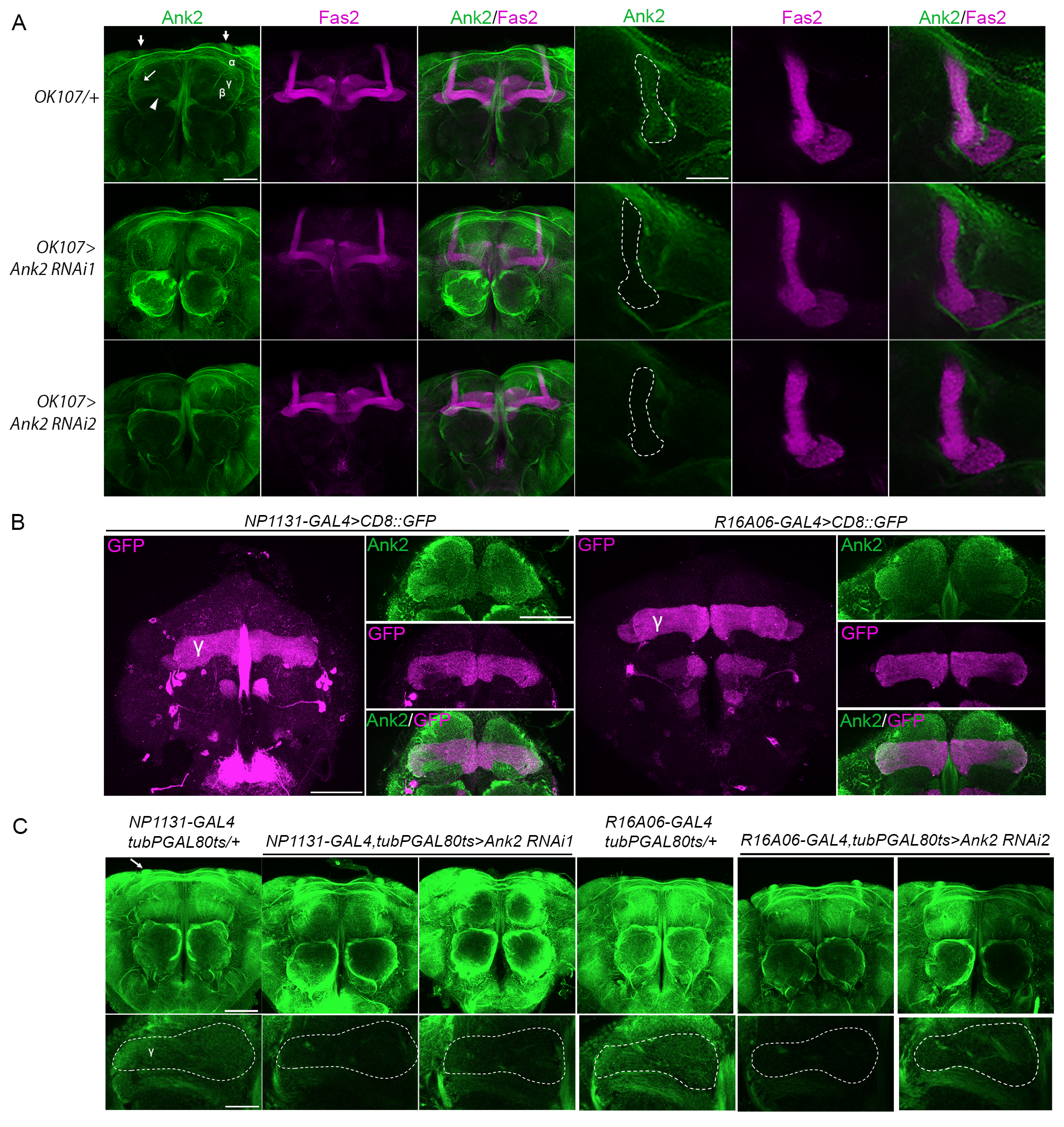

Supplement: Supplementary file 1 — Fig S1. Characterisation of Ank2 knockdown in the adult mushroom body. Inverted repeat hairpins for RNAi knockdown of Ank2 were expressed in in the mushroom body with OK107-GAL4. A. Confocal projection of brains labelled with Ank2-L (green) are shown, which are co-labelled with Fas2 (magenta), to highlight the mushroom body. Since Ank2 knockdown results in defects in lobe morphogenesis in some cases (Table 1), brains were selected in which the α and β lobes were intact in order to verify knockdown. Images in the three left columns are Z-stacks of 1 μm optical sections through the mushroom body. Ank2 is detected in the α, β and γ lobes of the mushroom body of control (OK107/+) brains. Short arrows point to the tips of the α lobe, the longer thinner arrow points to the γ lobe, and the arrowhead points to the β lobe. Expression in these lobes is absent in OK107>Ank2 RNAi1 and OK107>Ank2 RNAi2 mushroom bodies. Scale bar = 50 μm. As the expression in the mushroom body is somewhat obscured by other axon tracts, the three right columns show single 1 μm sections through the α lobe (outlined by a dashed line), which confirm knockdown with both Ank2 RNAi1 and RNAi2. Scale bar = 25 μm. B. The expression patterns of the γ lobe drivers R16A06-GAL4 and NP1131-GAL4 driving CD8::GFP, a plasma membrane-targeted GFP which allows for visualization of GFP in neuronal processes, confirm co-distribution with Ank2 in the γ lobe. The larger images are maximum projections through the mushroom body that show the expression patterns of each driver, with robust expression in the γ but not the α and β lobes (scale bar = 50 μm). Single 1 μm optical sections through the γ lobe labelled with Ank2 (green) and GFP (magenta) show expression of Ank2 in the γ lobes (scale bar = 25 μm). C. Knockdown with R16A06-GAL4 and NP1131-GAL4 reduces expression of Ank2 in the γ lobe. Ank2 staining appears overexposed in the Z-projections as it was optimized for detection of the γ lobe in the controls and the setti [file 13041_2023_1026_MOESM1_ESM.png]

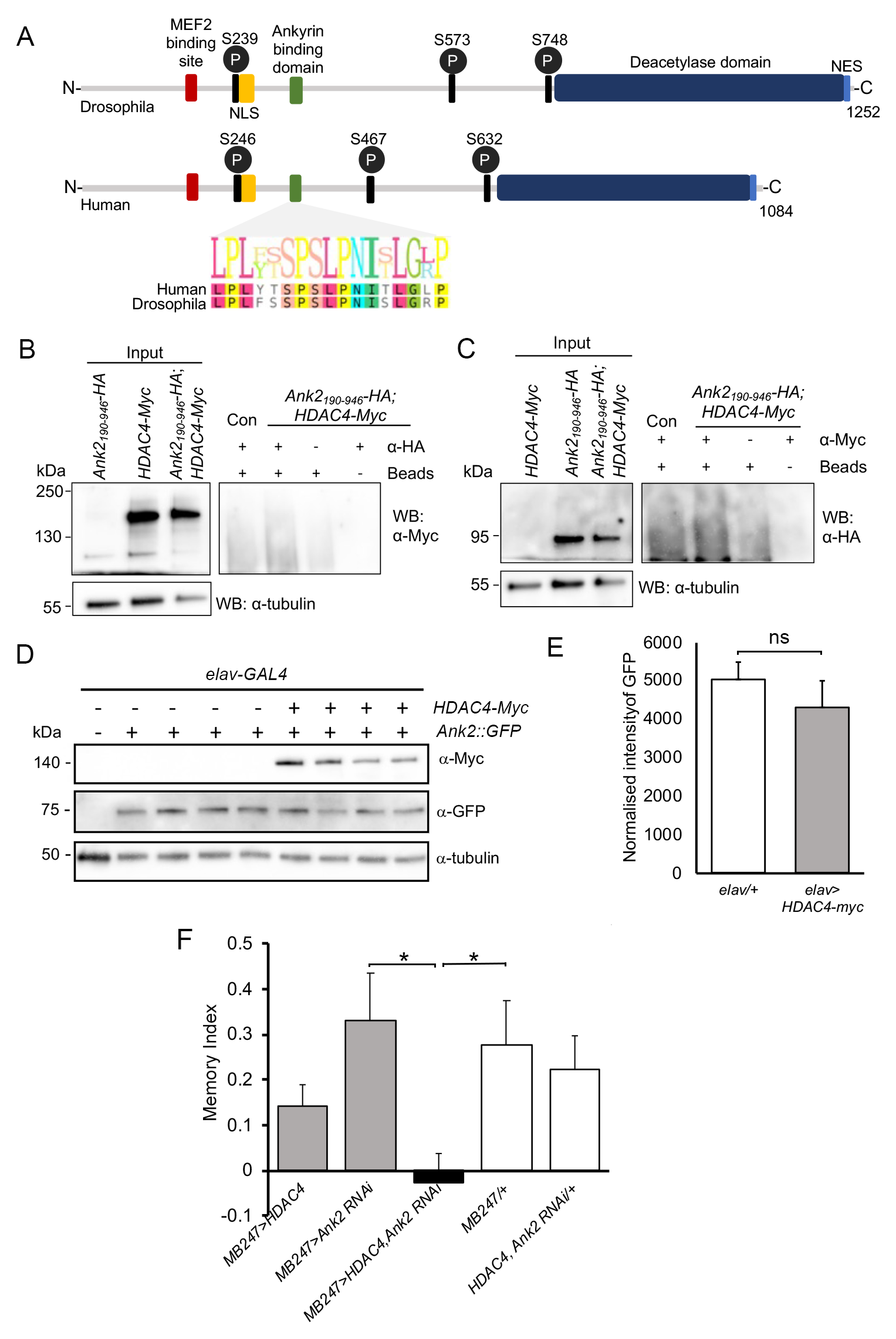

Supplement: Supplementary file 2 — Fig S2. Ank2 does not bind HDAC4 nor is its expression regulated by HDAC4. A. Domain structure of HDAC4 showing binding sites conserved between Drosophila and human HDAC4. The amino acid sequence of the region containing the PSLPNI ankyrin repeat motif that binds RFXANK and ANKRA2 in human HDAC4 is shown, with the corresponding amino acid sequence in Drosophila HDAC4. NLS, nuclear localisation sequence, NES, nuclear export sequence. Ps circled in black are serine residues that when phosphorylated provide binding sites for 14-3-3 mediated nuclear export. B,C. Co-immunoprecipitation of Ank2190-946-HA and HDAC4-Myc from whole cell lysates of fly heads expressing elav-GAL4 driven UAS-HDAC4-Myc and/or UAS-Ank2190-946-HA, as indicated with either anti-Myc or anti-HA. The estimated size of HDAC4-Myc is 144 kDa and Ank2190-946-HA is 95 kDa. Both blots were probed with anti-tubulin as a loading control. Input samples = 30 μg. B. Following immunoprecipitation with anti-HA, HDAC4-Myc was not detected upon probing with anti-Myc. C. In the reciprocal experiment, flies expressing Ank2190-946-HA; HDAC4-Myc were subjected to IP with anti-Myc, however Ank2190-946-HA was not detected upon probing with anti-HA. D. The effect of HDAC4 overexpression on the expression of Ank2 was examined. Ank2::GFP is a protein trap in which an exogenous GFP exon is inserted in frame into intron 1 of the endogenous Ank2 gene [62], resulting in GFP-tagging of a short isoform of Ank2 of approximately 75 kDa. elav-GAL4; Ank2::GFP flies were crossed to w(CS10) and UAS-HDAC4-Myc flies and whole head lysates of progeny were generated for western blotting. Samples were processed from four independent crosses. Blots were probed with anti-Myc to verify expression of HDAC4-Myc, and anti-GFP to determine whether the amount of Ank2::GFP normalized to tubulin is altered in the presence of HDAC4. E. There was no significant change in the level of Ank2::GFP on expression of HDAC4-Myc. F. MB247-GAL4;tubPGAL80ts was us [file 13041_2023_1026_MOESM2_ESM.png]
